# Editorial: Alloimmune Response From Regenerative Medicine

**DOI:** 10.3389/fimmu.2018.03121

**Published:** 2019-01-18

**Authors:** Reem Al-Daccak, Dominique Charron

**Affiliations:** ^1^Institut National de la Santé et de la Recherche Médicale (INSERM) UMRS-976, Université Paris-Diderot, Hôpital Saint-Louis, AP-HP, Paris and Labex Transplantex Unistra, Strasbourg, France; ^2^Shanghai Jiao Tong University, Shanghai, China

**Keywords:** stem cells, allogeneic, autologous, bioengineered tissues and organs, immuno-monitoring, stem cells derived extracellular vesicles/exosomes, stem cells repair and regeneration

In the second half of the twentieth century repair and regeneration of tissues and organs were initiated using transplantation procedures. Both autologous and allogenic approaches were undertaken, the later leading to an extensive body of basic, experimental, and translational research allowing the successful clinical development of transplantation medicine. This century has witnessed the hope that stem cells and bioengineered tissues and organs could provide valid alternatives to transplantation.

While stem cells therapy from autologous source could appear a logical and safer option to avoid detrimental immune response from the recipient, the absence of “off the shelf” immediate availability, complex logistics, comorbidities, and cost were considered strong limitations to their universal development. Allogenic stem cells appeared more pragmatic providing optimal “off the shelf” manufactured product for translation into the clinics in regenerative/reparative medicine. This promise was accentuated by evidence from ongoing research denoting that despite the conceptual questions of immunological barriers, allogeneic stem cells by controlling the local immune and inflammatory responses could enhance the paracrine regenerative effect. Within this scenario, allogeneic stem cell-based strategies would emerge as preferable over autologous one. We initiated this “Alloimmune Response from Regenerative Medicine” topic to provide a state-of-the-art evaluation of the potential risks and benefits of allogenic stem cell therapy toward facilitating translation into the clinics.

Reviewing and confirming the immunogenicity and immunomodulatory properties of allogeneic mesenchymal stem cells (MSC), Lohan et al. emphasized that more rigorous characterization and fine tuning of immunological behavior are warranted not only for MSC but also for their derived extracellular vesicles so that next generation strategies would hold promise for the treatment of multiple disease. To this end, the authors propose a set of immunology-based safety guidelines that would help a safer development of any allogenic stem cells and stem cells derived products. Evaluating the state-of-the-art in regard of immunogenicity and immune tolerance of pluripotent stem cells (PSCs) derivatives X. Liu and colleagues similarly confirmed the great therapeutic potential of PSCs-based strategies however within safe and effective tactics to induce graft-specific immune tolerance, mandatory for the clinic development of hiPSC-based therapy (Liu et al.). This would also apply for tissues and organs that are bioengineered with allogeneic hiPSC or human embryonic stem cells (hESC) where their potential immunogenicity is gaining relevance shifting the focus to the inherent control of immunogenicity to make these tissues/organs a clinical reality (1).

Within this perspective, R. Dressel and W.H. Zimmermann group proposed MHC-haploidentical parthenogenetic stem cells derived by pharmacological activation of unfertilized oocytes, as an alternative PSC-type allogenic source of therapeutic cells with less immunogenetic complexity. Through two interesting original research papers addressing the immune behavior of these cells and the cardiac reparative potential of their derived cardiomyocytes and engineered heart muscles (Didié et al.; Johannsen et al.), they provide a proof-of-concept within experimental mice models. Yet, despite their reduced immunogenic complexity related to MHC-restricted adaptive immune response, parthenogenetic stem cells express ligands for activating NK receptors and are therefore, similar to other PSC susceptible to activated natural killer cells cytotoxicity. While this susceptibility could increase the safety of transplanting relatively NK-resistant parthenogenic stem cell-derived cells, clinical translation of these cells, beyond their derived cardiomyocytes and engineered heart muscles, warrant further immunological fine tuning.

Lessons from highly regenerative organisms established that successful regeneration involves innate and adaptive immunity. Then, delivering regenerative therapies could rely on mastering the complex immune/inflammatory network of cells regulating tissue and wound homeostasis to “prepare the ground” for subsequent tissue repair. This premise is embraced by the “Allogeneic-driven-benefit” of cardiac stem/progenitor cells positioned by our studies of immunogenicity allogenicity in the context of cardiac regeneration and repair by allogeneic cardiac stem/progenitor cells (2), that we demonstrated able to master the immune/inflammatory network of cardiac injury and repair [(3, 4); Dam et al.]. The various immunological inputs driving a regeneration program are yet to be resolved, but the basic requirements for a regeneration-permissive immune environment are beginning to emerge.

The ongoing research argues that allogeneic strategies may lower the immunological response of the host cells by modulation or by regulatory mechanisms during the effector phase. This might designate “allogenicity” of therapeutic cells or cell-free products as key to regeneration and repair. Yet, Allogenicity is a striking example of a system that can produce both beneficial as well as detrimental effects, raising important conceptual, experimental and clinical issues. The potential humoral risk of stem cells [(5); Lohan et al.] stressed their allogenicity as a “Yin-Yang” opposing forces forming a dynamic system and called upon minimizing the risk while optimizing the benefit for an ultimate efficient allogeneic stem cell-based therapy. This would be even more important to avoid the immunization that would be detrimental if recipient of allogeneic stem cell therapy become later eligible for transplantation or if repeated administration of allogeneic cells is needed. Within this notion, the MHC/HLA emerges as a key player in basic, translational, and clinical aspects of regenerative medicine but also for logistics with reference to cell banks.

A systematic immuno-monitoring of donor-specific antibodies against HLA by Luminex-based assay would provide an immune-educated choice of the potential off-the-shelf allogeneic stem cells that might extend their persistence to activate the endogenous tissue regeneration and repair. Undeniably, whether stem cell-based or therapy or stem cell-bioengineered tissues/organs adequate immune-monitoring strategies need to be developed within the near future for efficacious clinical application (1, 5). Beyond the HLA system, other lessons learned in transplantation can also benefit the field of regeneration and repair. A new illustration comes from the work of Sarwal's group who identified using exomeSeq data new genetic variants outside the HLA system that discriminate donor and recipient of kidney transplantation for their long-term outcome particularly the incidence of antibody mediated rejection (Pineda et al.). The use of such predicting data prior transplantation could allow a better stratification of the patients and contribute to an optimal choice of D/R in transplant but also in stem cell-based therapy.

Overall, the learned consensus from the past and contemporary research is toward immune-educated new generation strategies to successful regenerative medicine. Stem cells are unique and time alone will tell whether allogeneic stem cell-based therapy could be supplanted by their derived extracellular vesicles/exosomes. Meanwhile, the current state-of-the-art might forecast the application of allogeneic stem cells followed by their cell-free extracellular vesicles/exosomes as a strategy that will not only elicit the cell-contact mediated reparative/regenerative immune response but also have the desired long-lasting effects through their generated products. Establishing the therapeutic value of such combinational strategy would probably provide a new dimension for regenerative medicine.

## Author Contributions

All authors listed have made a substantial, direct and intellectual contribution to the work, and approved it for publication.

### Conflict of Interest Statement

The authors declare that the research was conducted in the absence of any commercial or financial relationships that could be construed as a potential conflict of interest.
